# Supplementary material for: Half-quantum vortices and walls bounded by strings in the polar-distorted phases of topological superfluid 3He
Source: Nat Commun. 2019 Jan 16;10:237. doi: 10.1038/s41467-018-08204-8 (PMC6335426; doi:10.1038/s41467-018-08204-8)
Supplement: Supplementary file 1 — Supplementary Information [file 41467_2018_8204_MOESM1_ESM.pdf]

Supplementary Information for: Half-quantum vortices and walls bounded by strings in the polar-distorted phases of topological superfluid ^{3}He

J.T. Mäkinen[1*], V.V. Dmitriev[2], J. Nissinen[1], J. Rysti[1], G.E. Volovik[1,3], A.N. Yudin[2], K. Zhang[1,4], and V.B. Eltsov[1]

[1]*Low Temperature Laboratory, Department of Applied Physics,*
*Aalto University, FI-00076 AALTO, Finland; *E-mail: jere.makinen@aalto.fi*
[2]*P. L. Kapitza Institute for Physical Problems of RAS, 119334 Moscow, Russia*
[3]*Landau Institute for Theoretical Physics, 142432 Chernogolovka, Russia.*
[4]*University of Helsinki, Department of Mathematics and Statistics, P.O. Box 68 FIN-00014, Helsinki, Finland*

(Dated: December 14, 2018)

SUPPLEMENTARY FIGURES

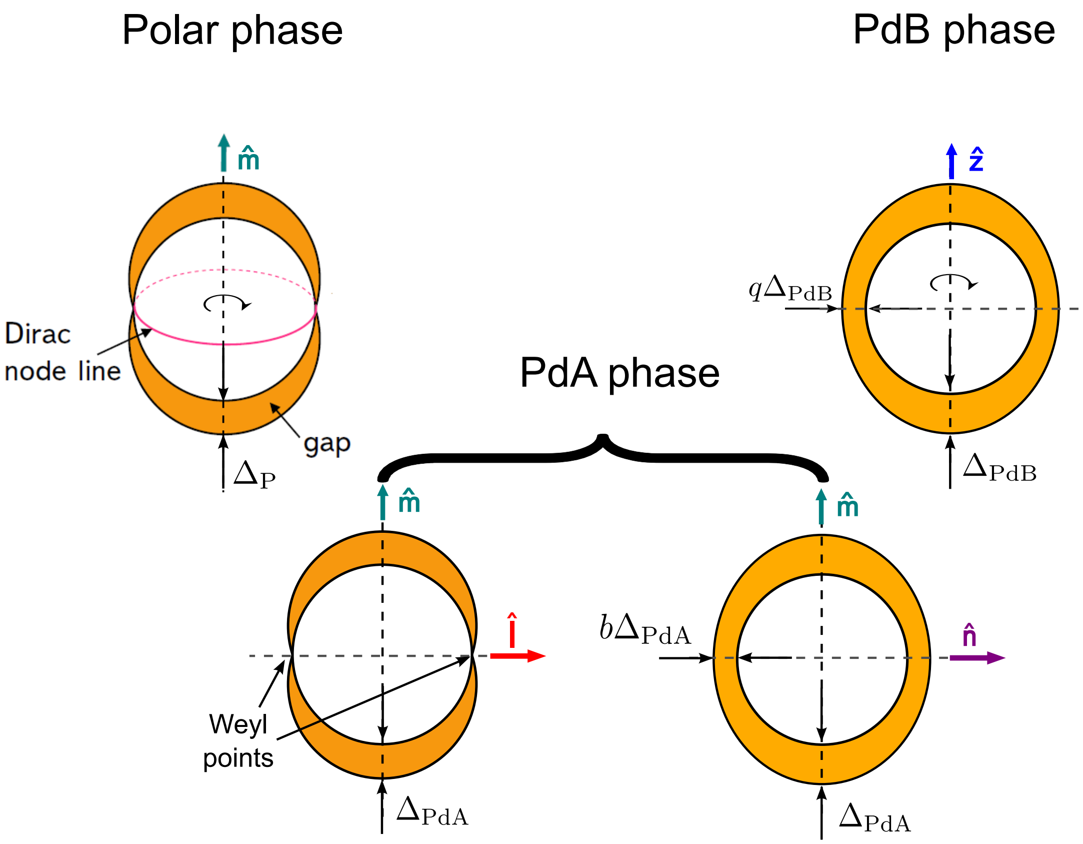

Supplementary Figure 1: Illustration of superfluid gaps in the polar-distorted phases. The figure shows schematic illustration (not to scale) of superfluid gaps in all superfluid phases encountered under confinement by nafen. The polar phase and PdB phase gaps are symmetric under rotation by the vertical axis, and the PdA phase gap is shown in two projections as it lacks the rotational symmetry.

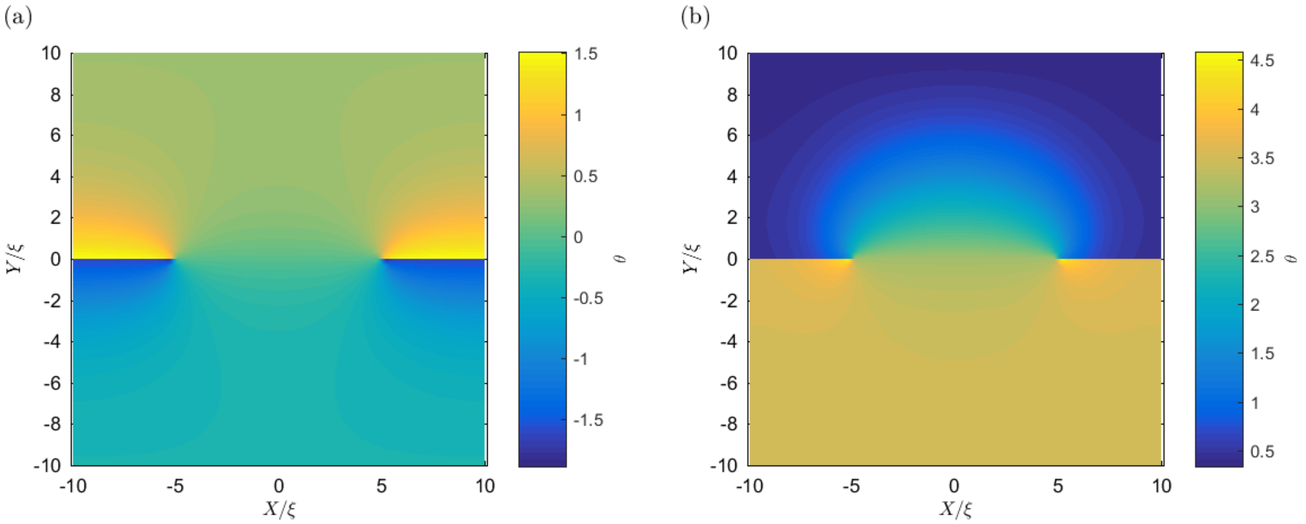

Supplementary Figure 2: 2D calculations of the order-parameter distribution in the vicinity of KLS walls. In both (a) and (b) panels the upper half corresponds to $q_2 > 0$ and the lower half to $q_2 < 0$. The KLS walls are located on the $y = 0$ axis between $X/\xi \in (-5, 5)$ and virtual jumps in the order parameter on the same axis between $X/\xi \in [-10, -5]$ and $X/\xi \in [5, 10]$. Plot (a) corresponds to the situation where the KLS walls and $(\pi - 2\theta_0)$-solitons are located between different HQV pairs. Plot (b) corresponds to the situation where the KLS Walls and $(\pi - 2\theta_0)$-solitons extend between the same HQV pair. Parameter value $q = 0.4$ was used in the calculations.

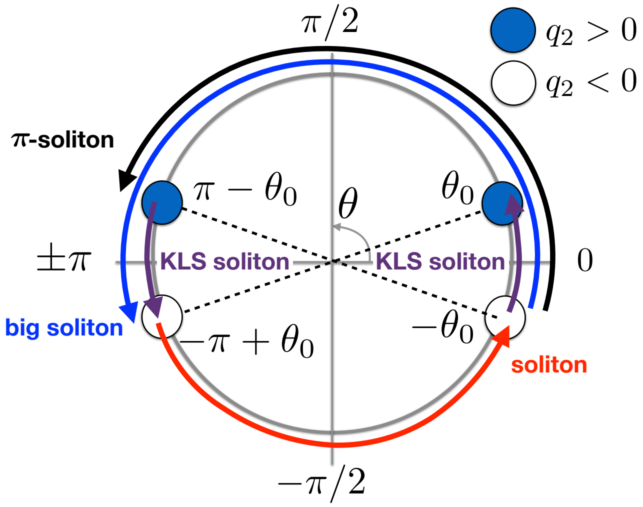

Supplementary Figure 3: Schematic illustration of the possible soliton solutions in the PdB phase. The blue circles correspond to the minima in Supplementary Eq. (23) for $q_2 > 0$ and white circles to $q_2 < 0$. Possible soliton solutions of the spin-winding angle θ of the order parameter [Supplementary Eq. (14)] are shown with arrows. The solitons are not symmetric under $\theta \to \theta + \pi$ ("small" and "big" solitons) and across the KLS wall, q_2 changes sign (the "KLS " and "π"-solitons).

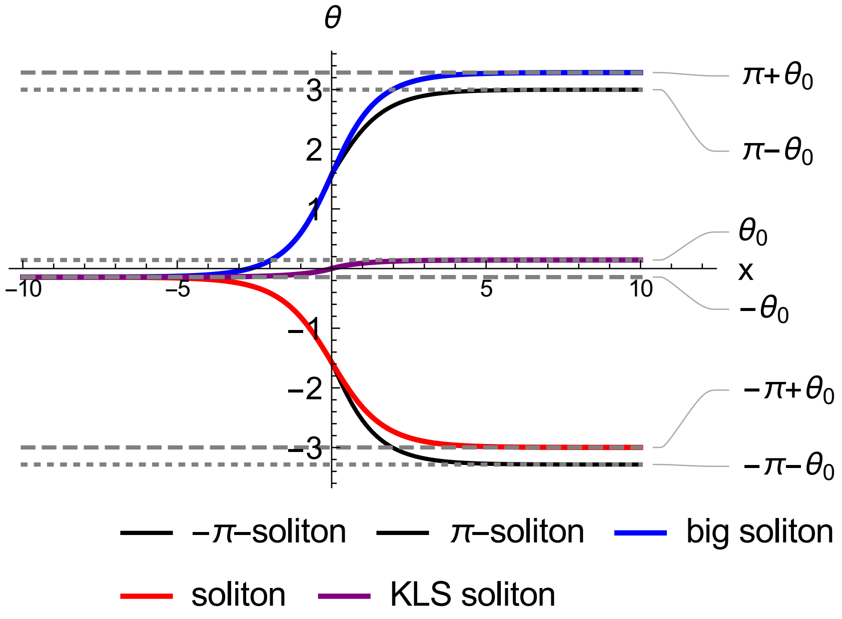

Supplementary Figure 4: 1D soliton solutions between various energy minima. The figure shows the 1D soliton solutions of Supplementary Eq. (22) for $q_2(-\infty) < 0$. The ordinary soliton has $\Delta\theta = \pi + 2|\theta_0|$ and $\theta(0) = -\pi/2$. The solution with $\theta(0) = \pi/2$ leads to the big soliton with winding $\Delta\theta = \pi - 2|\theta_0|$. Across the KLS wall, one must join the solutions with different signs of s_0 with $\Delta\theta = 2\theta_0$ or π. The latter is a composite of a big soliton and an ordinary soliton across the KLS wall.

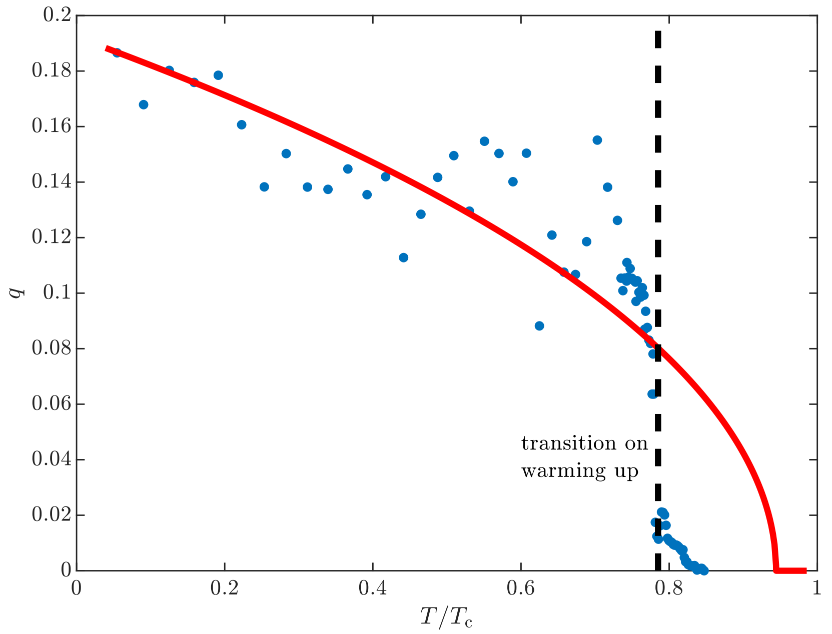

Supplementary Figure 5: Measured values of the distortion parameter on warming. The dots represent the measured values for q. The solid red line is an estimation of q, calculated based on Ginzburg-Landau theory with strong-coupling corrections using two fitting parameters in the spirit of Supplementary Ref. 12 and taking β-parameter values from Supplementary Ref. 23. The PdB phase critical temperature is shown for warming transition to the PdA phase.

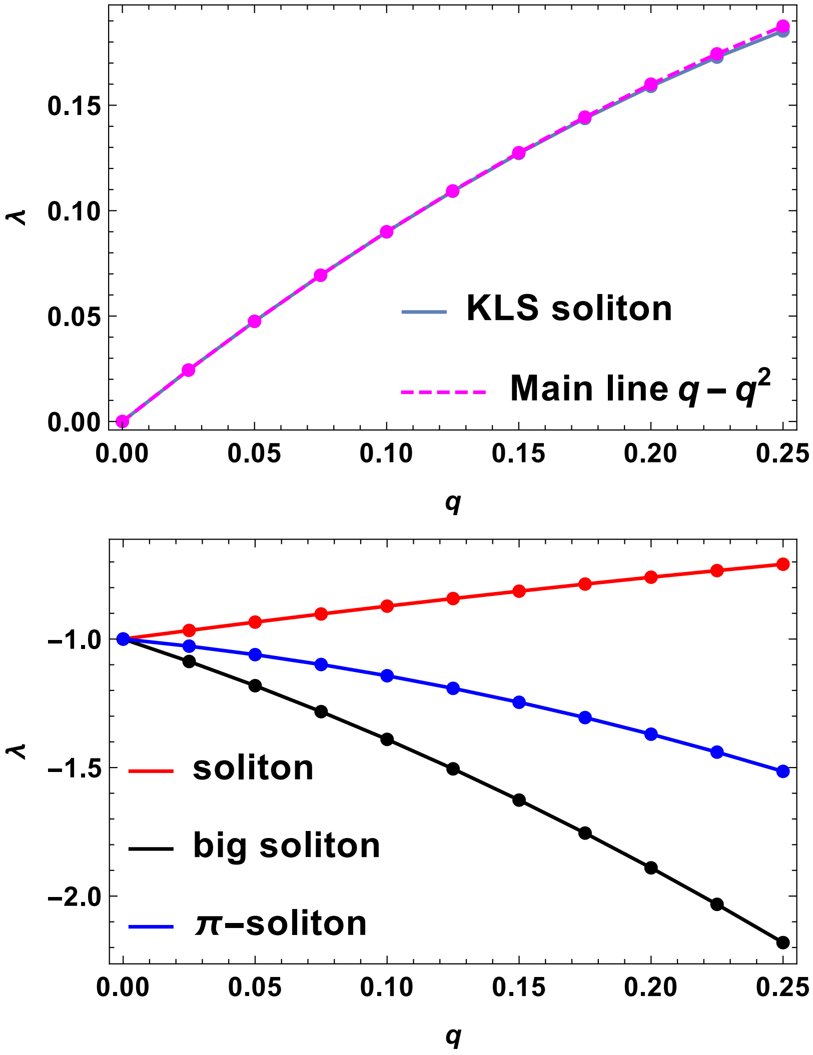

Supplementary Figure 6: Scaled frequency shifts for different soliton types in the PdB phase. The figures show the NMR resonance eigenvalue $\lambda(q)$ for spin waves localized on infinite 1D solitons. The frequency shift related the the KLS wall (upper figure) is indistinguishable from the frequency shift of the main line in the experimental range of q. The lower figure shows the frequency shifts for the other possible solitons as a function of q.

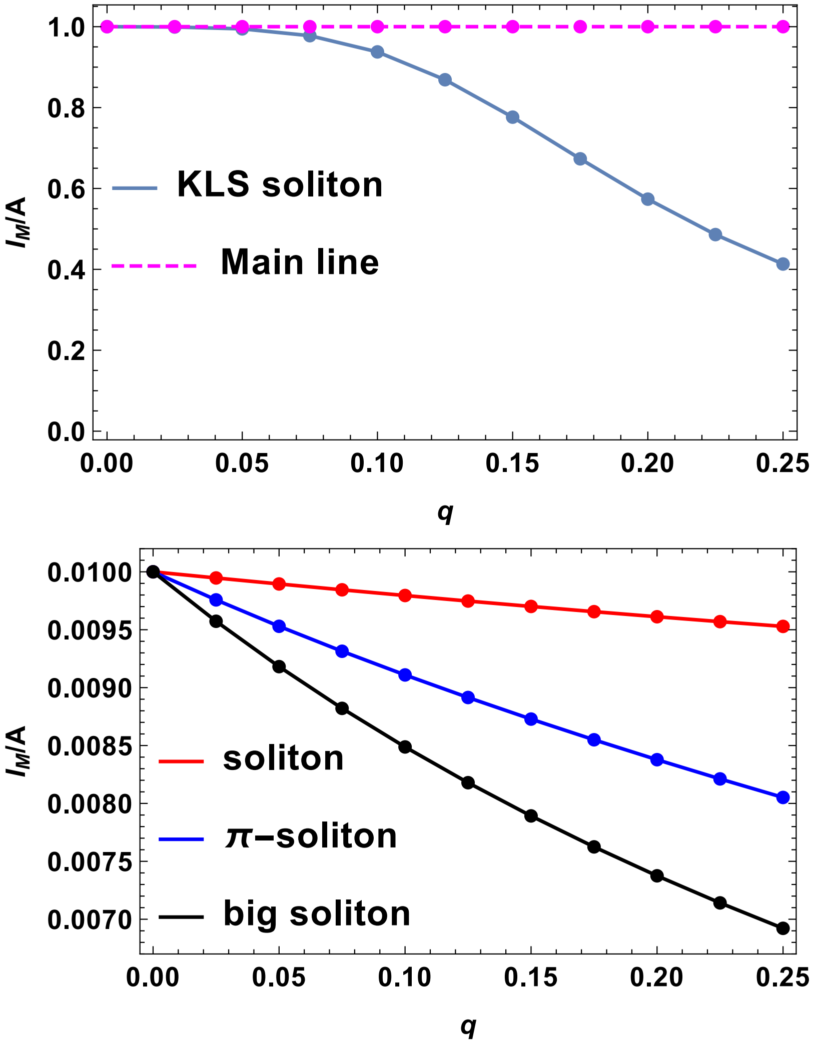

Supplementary Figure 7: Oscillator intensities as a function of distortion. The figures show the NMR oscillator intensities for spin waves on infinite 1D solitons. All solutions show decrease in the oscillator intensity, which results in the decrease of NMR satellite intensity – as observed in the experiments. However, the observed decrease in the intensity is much larger than the calculated decrease for realistic values of q.

SUPPLEMENTARY NOTE 1: SYMMETRIES OF LIQUID ^{3}HE IN CONSTRAINED GEOMETRY

Here we discuss the symmetries possessed by the normal fluid and the superfluid phases under confinement. For schematic illustration of the superfluid gaps in different phases, see Supplementary Figure 1.

Normal phase: Above the superfluid transition bulk ^{3}He possesses the symmetry group

$$G = SO(3)_L \times SO(3)_S \times U(1)_\phi \times T \times P \tag{1}$$

which includes continuous symmetries: three-dimensional rotations of coordinates $SO(3)_L$, rotations of the spin space $SO(3)_S$, and the global phase transformation group $U(1)_\phi$, as well as discrete symmetries; T is the time-reversal symmetry and P is the space parity symmetry. The transitions from normal fluid to superfluid phases as well as transitions between different superfluid phases are accompanied by the spontaneous breaking of continuous and/or discrete symmetries in G (in addition to the broken $U(1)_\phi$ symmetry of the superfluid). In bulk ^{3}He three superfluid phases can be realized; the fully-gapped superfluid B phase characterized by broken relative spin-orbit symmetry, the chiral $p_\mathrm{x} + ip_\mathrm{y}$ state known as the superfluid A phase, and finally, the spin-polarized A_1 phase in high magnetic fields.

In nanostructured confinement, i.e. in thin slabs[1,2] or in various aerogels,[3–7] the phase diagram, as well as the symmetry group of the normal phase, can be altered in a controlled fashion. In the presence of commercially available nematically ordered material called nafen,[5] the three-dimensional continuous rotational symmetry $SO(3)_L$ in Supplementary Eq. (1) is explicitly broken in the real space by the confinement. As a result, the total symmetry group of the normal phase is reduced to[8]

$$G' = D_{\infty L} \times SO(3)_S \times U(1)_\phi \times T \times P\,, \tag{2}$$

where $D_{\infty L}$ contains rotations about axis $\hat{\mathbf{z}}$ and π rotations about perpendicular axes. The resulting phase diagram[5] differs from that of the bulk ^{3}He; the critical temperature is suppressed and, more importantly, new superfluid phases, c.f. Supplementary Figure 1 - the polar, polar-distorted A (PdA), and polar-distorted B (PdB) phases - are observed.

Polar phase: In our samples, the phase transition with the highest critical temperature always occurs between the normal phase and the polar phase.[9] The order parameter of the polar phase can be written as

$$A_{\alpha j} = \frac{1}{\sqrt{3}} \Delta_\mathrm{P} e^{i\phi} \hat{\mathbf{d}}_\alpha \hat{\mathbf{m}}_j, \tag{3}$$

where $\Delta_\mathrm{P}(T)$ is the maximum superfluid gap in the polar phase, ϕ is the superfluid phase, $\hat{\mathbf{d}}$ is the unit vector of spin anisotropy, and $\hat{\mathbf{m}}$ is the unit vector of orbital anisotropy parallel to the anisotropy axis of the confinement. That is, in the transition to the polar phase the orbital part is fixed by the nafen strands and rotational symmetry is preserved only in the plane perpendicular to $\hat{\mathbf{m}}$. As for any superconducting or superfluid state, the phase acquires an expectation value and the phase gauge symmetry $U(1)_\phi$ is broken in the transition. The group describing the remaining symmetries of the polar phase in zero magnetic field is

$$H_\mathrm{P} = \tilde{D}_{\infty L} \times \tilde{D}_{\infty S} \times T \times \tilde{P}\,. \tag{4}$$

Here the discrete symmetry $\tilde{P}$ is the inversion P combined with the phase rotation $e^{\pi i}$. The symmetries $\tilde{D}_{\infty L}$ and $\tilde{D}_{\infty S}$ are the symmetries $D_{\infty L}$ and $D_{\infty S}$ in L and S spaces, where the π rotations about transverse axes are combined with a phase rotation $e^{\pi i}$. The homotopy group $\pi_1(G'/H_\mathrm{P}) = \mathbb{Z} \times \mathbb{Z}_2$ provides the topological stability of the phase vortices and the half-quantum vortex. The topological stability of spin vortices is determined by spin-orbit interaction and orientation of the magnetic field.[10]

Polar-distorted A phase: At certain nafen densities and pressures the polar phase transforms on cooling to the polar-distorted A (PdA) phase via a second-order phase transition.[5] The order parameter of the PdA phase is

$$A_{\alpha j} = \sqrt{\frac{1+b^2}{3}} \Delta_\mathrm{PdA} e^{i\phi} \hat{\mathbf{d}}_\alpha (\hat{\mathbf{m}}_j + ib\hat{\mathbf{n}}_j), \tag{5}$$

where the vector $\hat{\mathbf{n}}$ is an orbital anisotropy vector both perpendicular to vector $\hat{\mathbf{m}}$ and the Cooper pair orbital angular momentum axis $\hat{\mathbf{l}} = \hat{\mathbf{m}} \times \hat{\mathbf{n}}$, and $0 < b < 1$ is a dimensionless parameter characterizing the gap suppression by the confinement. The anisotropy vector $\hat{\mathbf{l}}$ defines the axis of the Weyl nodes in the PdA phase quasiparticle energy spectrum. The remaining symmetry group in the PdA phase in zero magnetic field is

$$H_\mathrm{PdA} = \tilde{D}_2 \times \tilde{D}_{\infty S} \times \tilde{P}\,. \tag{6}$$

The time-reversal symmetry is explicitly broken in the PdA phase, while $\tilde{P}$ combined with π orbital rotation about $\hat{\mathbf{m}}$ remains a symmetry. Together with π rotation about the axis $\hat{\mathbf{m}} \times \hat{\mathbf{n}}$ combined with the phase rotation $e^{\pi i}$ the orbital symmetry forms the $\tilde{D}_2$-group. The group H_PdA is the subgroup of H_P, which reflects the fact that the PdA phase can be obtained by the

second-order phase transition from the polar phase. The homotopy group $\pi_1(G'/H_{\rm PdA}) = \mathbb{Z}\times\mathbb{Z}\times\mathbb{Z}_2$ provides the topological stability of the phase vortices, the half-quantum vortex and also the orbital disclination in the vector $\hat{\mathbf{l}}$.

Polar-distorted B phase: The lowest temperature phase transition to the polar-distorted B phase (PdB) may in principle occur via a first-order transition from the PdA phase, or via a second-order phase transition from the polar phase. For the experimental conditions studied here, the transition occurs via a first-order phase transition. The order parameter of the PdB phase can be written as

$$A_{\alpha j} = \sqrt{\frac{1+2q^2}{3}}\Delta_{\rm PdB}e^{i\phi}(\hat{\mathbf{d}}_\alpha\hat{\mathbf{z}}_j + q_1\hat{\mathbf{e}}^1_\alpha\hat{\mathbf{x}}_j + q_2\hat{\mathbf{e}}^2_\alpha\hat{\mathbf{y}}_j)\,, \tag{7}$$

where $|q_1|, |q_2| \in (0,1)$, $|q_1| = |q_2| \equiv q$ describes the relative gap size in the plane perpendicular to the strands. Vectors $\hat{\mathbf{e}}^1$ and $\hat{\mathbf{e}}^2$ are unit vectors in the spin-space. The maximum gap $\Delta_{\rm PdB}(T,q)$ is achieved along the direction parallel to the strand orientation. For $q = 0$, we obtain the order parameter of the polar phase and for $q = 1$, we obtain the order parameter of the isotropic B phase. In zero magnetic field the total symmetry group describing the PdB phase can be written as

$$H_{\rm PdB} = D_{\infty J}\times T\times\tilde{P}\,, \tag{8}$$

where the notation J refers to the symmetry of the combined rotation of L and S simultaneously. The group $H_{\rm PdB}$ is again a subgroup of $H_{\rm P}$, which reflects the fact that the PdB phase can in principle be obtained by the second-order phase transition from the polar phase.

The homotopy group $\pi_1(G'/H_{\rm PdB}) = \mathbb{Z}\times\mathbb{Z}$ provides the topological stability of phase vortices and combined orbital and spin disclinations, but not of the half-quantum vortices. It is the lack of the last factor $\mathbb{Z}_2$ in the homotopy group which gives rise to the topologically unstable domain wall terminating on HQVs in the PdB phase: the KLS wall bounded by HQV strings.

SUPPLEMENTARY NOTE 2: FREE ENERGY OF THE POLAR-DISTORTED B PHASE

The Landau-Ginzburg free-energy of the PdB phase is given as (summation over repeated indices is assumed)

$$F = \int d^3x\, (f_{\rm grad} + f_{\rm bulk} + f_{\rm nafen} + f_{\mathbf{H}} + f_{\rm so}) \tag{9}$$

$$f_{\rm grad} = \tfrac{K_1}{2}(\nabla_i A_{\mu j})(\nabla_i A^*_{\mu j}) + \tfrac{K_2+K_3}{2}(\nabla_i A_{\mu i})(\nabla_j A^*_{\mu j}) \tag{10}$$

$$f_{\rm so} = g_{\rm so}\left(|\mathrm{Tr}(A)|^2 + \mathrm{Tr}(AA^*)\right) \tag{11}$$

$$f_{\rm nafen} = \tfrac{1}{2}\eta_{ij}A_{\mu i}A^*_{\mu j}, \quad \eta_{ij} = \eta\delta_{ij} + \Delta\eta\hat{\mathbf{z}}_i\hat{\mathbf{z}}_j, \tag{12}$$

$$f_{\mathbf{H}} = -\tfrac{1}{2}\mathbf{H}\boldsymbol{\chi}\mathbf{H}, \quad \chi_{\alpha\beta} = \chi_{\rm N}\delta_{\alpha\beta} - \tilde{\alpha}A_{\alpha i}A^*_{\beta i}, \tag{13}$$

where $f_{\rm grad}$ is the gradient energy, $f_{\rm bulk}$ is the standard bulk condensation energy of ^{3}He,[11] with order parameter matrix $A_{\mu i}$ corresponding to $d_{\mu i}$ in the notation of Supplementary Ref. 11. With this convention, the spin-orbit coupling corresponds to $g_{\rm so} = \frac{1}{5}\lambda_D N_{\rm F}$ of Supplementary Ref. 11. The effect of the nafen confinement $f_{\rm nafen}$, with the uniaxial anisotropy $\Delta\eta$ along $\parallel \hat{\mathbf{z}}$, is to renormalize the quadratic coefficients $\propto \Delta^2_{\rm PdB}$ since $T_{\rm c,nafen} < T_{\rm c}$ in bulk.[12] The magnetic susceptibility tensor $\boldsymbol{\chi}$ and the coefficient $\tilde{\alpha}$ for the PdB phase are also found in Supplementary Ref. 12.

The order parameter of polar distorted ^{3}He-B in nafen is parametrized by Supplementary Eq. (7). Here we concentrate on the limit of large polar anisotropy of the superfluid with the magnetic field $\mathbf{H} \neq 0$ transverse to the uniaxial anisotropy, i.e. the condition $|q_{1,2}| \ll 1$ holds. The corresponding ansatz for the spin part of the order parameter is

$$\begin{aligned}\hat{\mathbf{d}} &= R(\hat{\mathbf{y}},\theta)\hat{\mathbf{x}} = \cos\theta\hat{\mathbf{x}} - \sin\theta\hat{\mathbf{z}}\\ \hat{\mathbf{e}}^1 &= -R(\hat{\mathbf{y}},\theta)\hat{\mathbf{z}} = -\cos\theta\hat{\mathbf{z}} - \sin\theta\hat{\mathbf{x}}\\ \hat{\mathbf{e}}^2 &= \hat{\mathbf{y}}.\end{aligned} \tag{14}$$

We emphasize that with this parametrization $\hat{\mathbf{e}}^1 \times \hat{\mathbf{e}}^2 = \hat{\mathbf{d}}$ and the polar phase is obtained by setting $q = 0$, whereas the bulk B-phase corresponds to $q_1 = \pm q_2 = 1$. The degeneracy parameters for the bulk B phase are given by the rotation axis $\hat{\mathbf{n}} = \hat{\mathbf{y}}$ and angle $\cos\theta_{\hat{\mathbf{n}}} = \sin\theta$, which is the spin-orbit resolved Leggett angle.[11] To first order in $q_{1,2}$, the gradient energy is simply $f_{\rm grad} = \frac{1}{2}K^\theta_{ij}(\nabla_i\theta)(\nabla_j\theta)$ with $K^\theta_{ij} = K_1(\delta_{ij} - \hat{\mathbf{z}}_i\hat{\mathbf{z}}_j) + (K_1 + K_2 + K_3)\hat{\mathbf{z}}_i\hat{\mathbf{z}}_j$. With the ansatz [Supplementary Eq. (7)], the spin-orbit interaction takes the form

$$\begin{aligned}f_{\rm so} &= 2g_{\rm so}\Delta^2_{\rm PdB}\tilde{f}_{\rm so}(\theta),\\ \tilde{f}_{\rm so}(\theta) &\equiv (1+q_1)^2\sin^2\theta - (1+q_1)q_2\sin\theta - q_1 + q_2^2.\end{aligned} \tag{15}$$

SUPPLEMENTARY NOTE 3: FREE ENERGY OF SOLITONS AND DOMAIN WALLS

As discussed in the main text, the symmetry in the plane transverse to the anisotropy axis is broken by the magnetic field and/or the presence of defects. In equilibrium, $\hat{\mathbf{d}} \perp \mathbf{H}$ and we take $\hat{\mathbf{e}}^2 = \hat{\mathbf{y}}$ along $\mathbf{H}$. With this notation the KLS wall is a domain wall in q_2.

We now describe the order-parameter textures in the PdB phase which are associated with the HQVs pinned to the nafen strands. Similar, non-topological defects in the B-phase were already discussed in Supplementary Refs. 13 and 14, where the hierarchy of non-topological defects with length scales $\xi, \xi_{\|,\perp}$ (GL coherence lengths), ξ_D (dipole length) and ξ_H (magnetic healing length) was emphasized. More recently, non-topological defects with size ξ_D were termed "soft" and those of size ξ "hard" in Supplementary Ref. 15.

As discussed in the main text, the HQVs in the PdB phase are accompanied with a spin soliton and a KLS domain wall in the superfluid order parameter.

KLS walls: The KLS domain wall is the change of sign in the transverse, in-plane gap components $q_1\Delta_{\rm PdB}, q_2\Delta_{\rm PdB}$ determined by the in-plane coherence length $\xi_\perp$. Without loss of generality, we fix the domain wall to act only on $q_2\Delta_{\rm PdB}$ and the direction normal to the domain wall to be $\hat{\mathbf{x}}$. Let us write the bulk free energy of the PdB phase in nafen, Supplementary Eq. (9), as the sum of the polar phase free energy and the planar distortion $f_{\rm PdB} = f_{\rm P} + f_\perp$,

$$f_{\rm P} = \alpha_\| \Delta_{\rm PdB}^2 + \beta_{12345}\Delta_{\rm PdB}^4, \tag{16}$$

$$\begin{aligned} f_\perp &= (\alpha_\perp + 2\beta_{12}\Delta_{\rm PdB}^2)(q_1^2\Delta_{\rm dB}^2 + q_2^2\Delta_{\rm PdB}^2) \\ &\quad + 2\beta_{12}q_1^2\Delta_{\rm PdB}^2 q_2^2\Delta_{\rm PdB}^2 \\ &\quad + \beta_{12345}(q_1^4\Delta_{\rm PdB}^4 + q_2^4\Delta_{\rm PdB}^4), \end{aligned} \tag{17}$$

$$f_{\rm grad}[q_2\Delta_{\rm PdB}] = \tfrac{1}{2}K_{ij}^{(2)}(\nabla_i q_2\Delta_{\rm PdB})(\nabla_j q_2\Delta_{\rm PdB}), \tag{18}$$

where $K_{ij}^{(2)} = K_1(\delta_{ij} - \hat{\mathbf{y}}_i\hat{\mathbf{y}}_j) + (K_1 + K_2 + K_3)\hat{\mathbf{y}}_i\hat{\mathbf{y}}_j$. From $f_{\rm P}$ we obtain that $\Delta_{\rm PdB}^2 = -\alpha_\|/2\beta_{12345}$. For an infinite KLS wall along the y-axis, the order parameter is given by

$$\xi_{\perp 2}^2 \tfrac{d^2}{dx^2} q_2\Delta_{\rm PdB} = -q_2\Delta_{\rm PdB} + \tfrac{(q_2\Delta_{\rm PdB})^3}{(q_2\Delta_{\rm PdB}^0)^2}, \tag{19}$$

$$q_2(x)\Delta_{\rm PdB} = q_2\Delta_{\rm PdB}\tanh\left(\tfrac{x}{\xi_{\perp 2}}\right). \tag{20}$$

where $\xi_{\perp 2}^2/\xi_\|^2 \sim q^{-2} \gg 1$ and the KLS wall thickness is $q^{-1}\xi_\|$. For the KLS wall to be stable, this should be $\gg \xi_\|$, i.e. the distortion q should be small. However, on the length scale of the dipole length, $\xi_D^2 \sim K_1/g_{\rm so}$, relevant in NMR experiments, the KLS wall is thin, since $g_{\rm so} \ll -\alpha_\perp$. The free energy of the domain wall per unit area is

$$\sigma_{\rm KLS} \sim \xi_{\perp 2}\Delta f \sim -\xi_\| q f_\perp, \tag{21}$$

where $\Delta f \approx f_P - f_{\rm PdB}$. This surface tension makes the isolated HQVs unstable in the PdB phase without the nafen-pinning.[13,16]

Spin solitons: Spin solitons have thickness of the order of the dipole length ξ_D. The distribution of $\theta(\mathbf{r})$ in the presence of HQV spin solitons is found as a minimum of energy in Supplementary Eq. (9),

$$-\nabla_i\frac{\delta F}{\delta\nabla_i\theta} + \frac{\delta F}{\delta\theta} = -\xi_{D,ij}^2\nabla_i\nabla_j\theta(\mathbf{r}) + \tfrac{1}{2}\frac{\delta\tilde{f}_{\rm so}(\theta(\mathbf{r}))}{\delta\theta} = 0. \tag{22}$$

In bulk the energy is minimized for a homogeneous $\theta = \theta_0$ or $\pi - \theta_0$, where

$$\theta_0 = \arcsin\frac{q_2}{2(1+q_1)}. \tag{23}$$

The minima for the spin-orbit potential $f_{\rm so}(\theta)$ depend on the sign of q_2, which changes across the KLS walls. In contrast to the polar phase with $q_1 = q_2 = 0$, the potential is no longer symmetric under $\theta \to \theta + \pi$, see Supplementary Figure 3.

The equations can be solved analytically for the infinite soliton uniform in the y- and z-directions. Integrating Supplementary Eq. (22) over y and z we obtain

$$\xi_D^2(\theta')^2 = (1+q_1)^2(\sin\theta - \sin\theta_0)^2 + C, \tag{24}$$

where $C = 0$ by the bulk boundary conditions $\theta(x \to \pm\infty) = \theta_0$, $\theta'(x \to \pm\infty) = 0$. The soliton solutions are

$$\theta(\pm\tilde{x}) = \mp\pi/2 \mp 2\arctan f_\mp(\tilde{x}; s_0), \tag{25}$$

where we scaled $\tilde{x} \equiv (1+q_1)x/\xi_D$, abbreviated $s_0 \equiv \sin\theta_0$ and

$$f_{\mp}(\tilde{x}; s_0) = \sqrt{\tfrac{1\mp s_0}{1\pm s_0}}\tanh(\sqrt{1-s_0^2}\tilde{x}/2) \tag{26}$$

where there are two solutions corresponding to the two signs in Supplementary Eq. (24) and we have used the boundary conditions $\theta(0) = \{-\pi/2, +\pi/2\}$. The two soliton solutions in Supplementary Eq. (25) have windings $\pi \mp 2\theta_0$. Clearly the two solutions interchange as $s_0 \to -s_0$.

When $\xi \ll \xi_D$, we can approximate the KLS domain wall as $q_2(x) = q\mathrm{sign}(x)$. Across a KLS wall, $\theta_0 \to -\theta_0$ and $s_0 \to -s_0$ and we can respectively join the corresponding solutions with boundary conditions $\theta(0) = 0$ or $\pm\pi/2$ at the KLS wall, see Supplementary Figures 3 and 4. In particular, we can find a solution with $\Delta\theta = 2\theta_0$ that crosses $\theta(0) = 0$ and a solution with $\Delta\theta = \pi$, $\theta(0) = \pm\pi/2$ composed of a small and big soliton on the opposite sides of the domain wall. For $q_2 = \mathrm{sgn}(x)|q_2|$, the KLS soliton solution with $\Delta\theta = 2\theta_0$ is given by

$$\theta_{\mathrm{KLS}}(\tilde{x}) = 2\arctan\left(\frac{\mathrm{sgn}(x)|s_0|}{1+\sqrt{1-s_0^2}\coth(\sqrt{1-s_0^2}|\tilde{x}|/2)}\right). \tag{27}$$

The plots of the soliton solutions interpolating between the PdB spin-orbit energy minima in Supplementary Figure 3 are found in Supplementary Figure 4: In summary, we find two solitons (“soliton” and “big soliton”) without KLS wall and two solitons (the “ KLS soliton” and the “π-soliton”) connecting the solutions with opposite sign of s_0. In terms of the more realistic 2D HQV-pair structures depicted in Supplementary Figure 2 or in the main text, the separate 1D small soliton and KLS soliton roughly corresponds to the case shown in (a) in Supplementary Figure 2 with the KLS wall outside the spin soliton, whereas the π-soliton corresponds to that shown in Supplementary Figure 2 (b).

The free energy per unit area of a 1D spin soliton is

$$\begin{aligned}\sigma_{\mathrm{spin}} &= \frac{1}{LR_{\mathrm{HQV}}}\int d^3\mathbf{r}\,\left(f[\theta(\mathbf{r})] - f[\theta_0]\right) \\ &\approx \frac{\chi}{2\gamma^2}\Omega_{\mathrm{PdB}}^2\int dx\; 2(\tilde{f}_{\mathrm{so}}(\theta(x)) - \tilde{f}_{\mathrm{so}}(\theta_0)) \sim \xi_D\Delta f_{\mathrm{soliton}}.\end{aligned} \tag{28}$$

where L is the linear size along z-direction (the height of the sample), and R_{HQV} the the linear size along y-direction (the distance between HQVs bounding the soliton).

SUPPLEMENTARY NOTE 4: PINNING OF HQV BY A COLUMNAR DEFECT

Let us consider what happens with HQVs, when the phase transition is crossed between polar phase and the PdB phase. Our experiments demonstrate that if originally the polar phase contains pinned HQVs, they survive the transitions to the PdB phase and back to the polar phase. From this one can conclude that the HQVs remained pinned even after the formation of a KLS domain wall formed between two HQVs, demonstrating that HQVs are so strongly pinned that the tension of the KLS wall can not unpin vortices. Let us consider the pinning in more detail (assuming $\hbar = 1$ and $k_{\rm B} = 1$).

The radius of the columnar defect – the nafen strand – is small compared to the coherence length in superfluid ^{3}He. According to Supplementary Ref. 17 the characteristic energy of the order parameter distortion produced by a mesoscopic object of size $R < \xi_0 \equiv \xi(T=0)$ is (per unit length of the cylinder):

$$E_{\rm P} \sim k_{\rm F}^2 R \frac{\Delta^2}{T_{\rm c}} \ , \ R < \xi_0 \, , \tag{29}$$

where $k_{\rm F}$ is the Fermi momentum, $\Delta \sim v_{\rm F}\xi^{-1}$ is the superfluid gap (here we use general gap notation, since this is an order-of magnitude estimation and $\Delta \sim \Delta_{\rm PdB}$), and $v_{\rm F}$ is the Fermi velocity. This equation was used in particular for the estimation of the orientational energy of the nafen strands on the orbital vector $\hat{\mathbf{l}}$ in ^{3}He-A in relation to the Larkin-Imry-Ma effect.[18] The Larkin-Imry-Ma effect due to the random anisotropy produced by the random orientation of strands was observed later.[19,20]

Supplementary Eq. (29) can be applied for the pinning of the texture $\Delta(\mathbf{r})$ by columnar defects – the nafen strands. The pinning force comes from the coordinate dependence of the energy of the columnar object in the texture: $F_{\rm P} \sim \nabla E_{\rm P}$. For textures with characteristic length scale ξ, one has $F_{\rm P} \sim \nabla E_{\rm P} \sim E_{\rm P}/\xi$. For vortices, including the half-quantum vortices observed in Supplementary Ref. 21, the pinning force from the columnar defect of radius R is (assuming $\Delta/T_{\rm c} \sim \xi_0/\xi$):

$$F_{\rm P} \sim k_{\rm F}^2 v_{\rm F} \frac{R}{\xi^2}\frac{\Delta}{T_{\rm c}} \sim k_{\rm F}^2 v_{\rm F} \frac{R\xi_0}{\xi^3} \ , \ R < \xi_0 \, . \tag{30}$$

Let us compare the pinning force with the tension of the KLS wall of thickness $\xi_{\rm W} \sim q^{-1}\xi \gg \xi$, given by

$$F_{\rm KLS} \sim k_{\rm F}^2 v_{\rm F} \frac{q^2}{\xi_{\rm W}}\frac{\Delta^2}{T_{\rm c}^2} \sim k_{\rm F}^2 v_{\rm F} q^3 \frac{\xi_0^2}{\xi^3} \, . \tag{31}$$

The tension from the KLS wall can not unpin the HQV if $F_{\rm KLS} < F_{\rm P}$, or if

$$q^3 < \frac{R}{\xi_0} < 1 \, . \tag{32}$$

Close to the transition from the polar to PdB phase, the HQVs remained pinned, while the KLS wall is pinned by the pinned HQVs.

Let us consider the pinning force by the columnar defect for different ranges of R. For $R > \xi$ the pinning does not depend on R, but instead is given by the characteristic length scale ξ. The dependence of the pinning force on R is given by

$$\frac{F_{\rm P}}{k_{\rm F}^2 v_{\rm F}} \sim \frac{R\xi_0}{\xi^3} \ , \ R < \xi_0 \, , \tag{33}$$

$$\frac{F_{\rm P}}{k_{\rm F}^2 v_{\rm F}} \sim \frac{R^2}{\xi^3} \ , \ \xi_0 < R < \xi \, , \tag{34}$$

$$\frac{F_{\rm P}}{k_{\rm F}^2 v_{\rm F}} \sim \frac{1}{\xi} \ , \ R > \xi \, . \tag{35}$$

SUPPLEMENTARY NOTE 5: SPIN WAVES AND NMR IN THE PDB PHASE

We study the HQVs and KLS domain walls in the PdB phase via their influence on the NMR spin-wave spectrum through the order parameter textures of solitons. The relevant Hamiltonian is given by the magnetic field energy and the superfluid spin degrees of freedom in the London limit,

$$\mathbf{H} = \frac{1}{2}\gamma^2 \mathbf{S}\chi^{-1}\mathbf{S} - \gamma \mathbf{H}\cdot\mathbf{S} + f_{\mathrm{grad}} + f_{\mathrm{so}}, \tag{36}$$

where $\mathbf{S}$ is the total spin density, γ is the gyromagnetic ratio of ^{3}He and χ is the principal axis of the magnetic susceptibility tensor along $\hat{\mathbf{d}}$. The Leggett equations for the spin $\mathbf{S}$ and the order parameter spin-triad $\hat{\mathbf{e}}^I = \{\hat{\mathbf{e}}^1, \hat{\mathbf{e}}^2, \hat{\mathbf{d}}\}$, where $I = 1, 2, 3$, are

$$\partial_t \mathbf{S} = \{\mathbf{S}, \mathbf{H}\} = \gamma \mathbf{S}\times\mathbf{H} + \tfrac{\delta(f_{\mathrm{grad}}+f_{\mathrm{so}})}{\delta \hat{\mathbf{e}}^I}\{\mathbf{S}, \hat{\mathbf{e}}^I\} \tag{37}$$

$$\partial_t \hat{\mathbf{e}}^I = \{\hat{\mathbf{e}}^I, \mathbf{H}\} = -\frac{\gamma^2}{\chi}\hat{\mathbf{e}}^I \times \delta\mathbf{S}, \tag{38}$$

with the semiclassical Poisson brackets $\{S_\alpha, S_\beta\} = \epsilon_{\alpha\beta\gamma}S_\gamma$ and $\{S_\alpha, e^I_\beta\} = \epsilon_{\alpha\beta\gamma}e^I_\gamma$. In this parametrization, the spin-orbit interaction [Supplementary Eq. (11)] takes the form

$$f_{\mathrm{so}}[\hat{\mathbf{e}}^I] = 2g_{\mathrm{so}}\Delta^2_{\mathrm{PdB}}\left(\hat{\mathbf{e}}^I\cdot\mathbf{B}^{IJ}\cdot\hat{\mathbf{e}}^J\right) \tag{39}$$

where $\mathbf{B}^{IJ} = \frac{1}{2}(\delta^{IM}\delta^{JN} + \delta^{IN}\delta^{JM})\tilde{\mathbf{r}}^M\tilde{\mathbf{r}}^N$ is a matrix in orbital space $\tilde{\mathbf{r}}^M = \{q_1\hat{\mathbf{x}}, q_2\hat{\mathbf{y}}, \hat{\mathbf{z}}\}$ defined by the orbital part of the order parameter and summation over repeated spin-triad and orbital indices $I, J = 1, 2, 3$ and $M, N = 1, 2, 3$ is implied, respectively.

We look for solutions in small oscillations to linear order around an equilibrium state $\frac{\delta H}{\delta \mathbf{S}_0} = \frac{\delta H}{\delta \hat{\mathbf{e}}^I_0} = 0$. Eliminating $\delta\hat{\mathbf{e}}^I$ from the system of Leggett equations, we arrive to

$$\omega^2\delta\mathbf{S} = i\omega\omega_{\mathrm{L}}(\hat{\mathbf{H}}\times\delta\mathbf{S}) + \Omega^2_{\mathrm{PdB}}\mathbf{\Lambda}\cdot\delta\mathbf{S}, \tag{40}$$

where we have defined the "Leggett frequency" of the PdB phase as the quantity

$$\Omega^2_{\mathrm{PdB}} = 4g_{\mathrm{so}}\gamma^2\Delta^2_{\mathrm{PdB}}/\chi_{\mathrm{PdB}} \tag{41}$$

which we stress is *not* equal to the longitudinal NMR frequency of the PdB phase for $q_1, q_2 \neq 0, 1$, see below. The matrix $\mathbf{\Lambda} = \mathbf{\Lambda}^{\mathrm{grad}} + \mathbf{\Lambda}^{\mathrm{so}}$ is defined by

$$\Lambda^{\mathrm{grad}}_{\alpha\beta} = \xi^2_{D,ij}\Big((\delta_{\alpha\beta} - \hat{\mathbf{d}}^0_\alpha\hat{\mathbf{d}}^0_\beta)\nabla_i\nabla_j + \hat{\mathbf{d}}^0_\alpha(\nabla_i\nabla_j\hat{\mathbf{d}}^0_\beta) - \hat{\mathbf{d}}^0_\beta(\nabla_i\nabla_j\hat{\mathbf{d}}^0_\alpha) - 2\hat{\mathbf{d}}^0_\beta(\nabla_i\hat{\mathbf{d}}^0_\alpha)\nabla_j\Big) \tag{42}$$

$$\Lambda^{\mathrm{so}}_{\alpha\beta} = e^I_{0\alpha}B^{IJ}_{\beta\delta}e^J_{0\delta} - \tilde{e}^I_{0\nu}B^{IJ}_{\nu\delta}e^J_{0\delta}\delta_{\alpha\beta} + \epsilon_{\alpha\nu\gamma}\epsilon_{\delta\mu\beta}e^I_{0\gamma}B^{IJ}_{\nu\delta}e^J_{0\mu}, \tag{43}$$

where $B^{IJ}_{\alpha\beta}$ is the matrix in Supplementary Eq. (39) for each I, J, $\xi^2_{D,ij} = K^\theta_{ij}/4g_{\mathrm{so}}$ and the gradient energy is taken to first order in q_1, q_2. In the limit $q_1 = q_2 = 0$, the equations are those of the polar phase;[22] in particular the lowest order $\mathbf{\Lambda}^{\mathrm{grad}}$ in Supplementary Eq. (42) coincides with the expression for the polar phase given in Supplementary Ref. 5.

With $\mathbf{H} \parallel \hat{\mathbf{y}}$ and within the approximation $\omega \approx \omega_{\mathrm{L}}$ to lowest order in $\frac{\Omega_{\mathrm{PdB}}}{\omega_{\mathrm{L}}} \ll 1$, Supplementary Eq. (40) separates for transverse $\delta S_+ = (\delta S_z + i\delta S_x)/\sqrt{2}$ and longitudinal spin waves as[22]

$$\tfrac{\omega^2-\omega_L^2}{\Omega^2_{\mathrm{PdB}}}\delta S_+ = (\Lambda_{xx} + \Lambda_{zz})\delta S_+ + i(\Lambda_{xz} - \Lambda_{zx})\delta S_+, \tag{44}$$

$$\tfrac{\omega^2}{\Omega^2_{\mathrm{PdB}}}\delta S_y = \Lambda_{yy}\delta S_y. \tag{45}$$

Transverse magnetic field: For the transverse orientation of the magnetic field to the uniaxial nafen anisotropy along $\hat{\mathbf{z}}$, the order parameter is given by Supplementary Eq. (7). The transverse spin wave equation becomes

$$-\tfrac{\omega^2-\omega_{\mathrm{L}}^2}{\Omega^2_{\mathrm{PdB}}}\Psi_+ = \xi^2_{D,ij}\Big(\nabla_i\nabla_j + (\nabla_i\theta)(\nabla_j\theta)\Big)\Psi_+ + \Big(\tilde{f}_{\mathrm{so}}(\theta) - \tfrac{3}{2}(1+q_1)q_2\sin\theta + q_2^2\Big)\Psi_+ \tag{46}$$

where $\Psi_+ = e^{i\theta}\delta S_+$, the dimensionless spin-orbit interaction $\tilde{f}_{\rm so}(\theta)$ is defined in Supplementary Eq. (15) and the longitudinal spin wave equation is

$$-\frac{\omega^2}{\Omega^2_{\rm PdB}}\delta S_y = \xi^2_{D,ij}\nabla_i\nabla_j\delta S_y - \left(\frac{\partial^2 \tilde{f}_{\rm so}}{\partial\theta^2}\right)\delta S_y. \tag{47}$$

The transverse frequency shift with uniform $\theta = \theta_0$ (i.e. the response of the bulk) is given as

$$\frac{\omega^2_\perp - \omega^2_{\rm L}}{\Omega^2_{\rm PdB}} = q_1 - q_2^2. \tag{48}$$

This frequency shift was reported also in Supplementary Ref. 12.

Axial field: In axial field, i.e. $\mathbf{H}$ along the uniaxial anistropy, the order parameter is no longer given by Supplementary Eq. (7). Since the magnetic field energy is dominating and $\hat{\mathbf{d}} \perp \mathbf{H} \parallel \hat{\mathbf{y}}$ which is also the direction of the uniaxial anisotropy (and not along $\hat{\mathbf{z}}$ as in the preceding sections), we parametrize the orbital part of order parameter as

$$A_{\mu i} = \Delta_{\rm PdB}e^{i\phi}\left(\hat{\mathbf{d}}_\mu\hat{\mathbf{y}}_i + q_1\hat{\mathbf{e}}^1_\mu\hat{\mathbf{x}}_i + q_2\hat{\mathbf{e}}^2_\mu\hat{\mathbf{z}}_i\right), \tag{49}$$

whereas the spin part is still given by Supplementary Eq. (14) with the rotation angle in the plane perpendicular to the magnetic field. The spin-orbit interaction takes the form $\tilde{f}_{{\rm so},\parallel}(\theta) = q_1^2\sin^2\theta - q_2\sin\theta$ with $\sin\theta_{0,\parallel} = 0$ for $|q| < 1/2$.[12] The longitudinal spin wave equation in axial field follows from Supplementary Eq. (47) with this replacement.

The transverse spin wave $\Psi_+ = e^{i\theta}\delta S_+$ equations are given as

$$\begin{aligned}-\frac{\omega^2_\parallel-\omega^2_{\rm L}}{\Omega^2_{\rm PdB}}\Psi_+ &= \xi^2_{D,ij}\Big(\nabla_i\nabla_j + (\nabla_i\theta)(\nabla_j\theta)\Big)\Psi_+ \\ &+\Big(-1-\tfrac{5}{2}q_2\sin\theta + q_1^2\sin^2\theta - q_2^2\Big)\Psi_+.\end{aligned} \tag{50}$$

The homogeneous transverse frequency shift in axial field with uniform $\theta = \theta_{0,\parallel} = {\rm sgn}(q_2)\pi/2$ is given as

$$\frac{\omega^2_\parallel - \omega^2_{\rm L}}{\Omega^2_{\rm PdB}} = 1 + \frac{5}{2}|q_2|, \tag{51}$$

which is equal to the value reported in Supplementary Ref. 12.

SUPPLEMENTARY NOTE 6: DETERMINATION OF THE DISTORTION PARAMETER q

The q-parameter value is determined from the frequency shifts in Supplementary Eqs.(48) and (51), following a method described in Supplementary Ref. 12. In the experimental region of interest, the distortion factor $q = q_1 = |q_2|$ is

$$q = \frac{2-5C}{4} - \frac{1}{4}\sqrt{25C^2 - 36C + 4}, \tag{52}$$

where $C = (\omega_\perp - \omega_\mathrm{L})/(\omega_\| - \omega_\mathrm{L})$. The expression [Supplementary Eq. (52)] is valid in the range $q \in [0, (\sqrt{14} - 2)/5]$. We carefully prepare the state by cooling the sample through the superfluid transition temperature in zero rotation in transverse magnetic field to avoid creation of half-quantum vortices. Then we cool the sample down to the lowest temperatures and start warming it up slowly, continuously monitoring the NMR resonance spectrum either in axial or transverse field. This way we can measure the q-parameter in the coexistence region of the PdA and PdB phases. The results of our measurements are shown in Supplementary Figure 5.

SUPPLEMENTARY NOTE 7: SPIN WAVES ON 1D SOLITONS AND KLS WALLS

The transverse spin wave equation [Supplementary Eq. (46)] takes the form of an eigenvalue equation

$$\lambda\Psi_+ = -\nabla_i\nabla_j\Psi_+ - U(\theta(\mathbf{r}))\Psi_+ \tag{53}$$

with eigenvalue $\lambda \equiv \frac{\omega^2-\omega_{\rm L}^2}{\Omega_{\rm PdB}^2}$ and potential

$$U(\theta) = -\xi_{D,ij}\nabla_i\theta\nabla_j\theta - \tilde{f}_{\rm so}(\theta) + \tfrac{3}{2}(1+q_1)q_2\sin\theta - q_2^2. \tag{54}$$

The spin-wave spectrum is therefore defined with respect to the order parameter texture $\theta(\mathbf{r})$, as determined by the GL equations in Supplementary Eq. (22). The homogeneous δS_+ excitation (the main NMR line) is shifted from the Larmor value by $\Delta\omega \equiv \omega - \omega_{\rm L} \approx \frac{\Omega_{\rm PdB}^2}{2\omega_{\rm L}}\lambda_{\theta_0}$ with $\lambda_{\theta_0} = U(\theta_0)$. As discussed, the solutions $\theta(x)$ are analytically tractable in 1D and the spin wave equation [Supplementary Eq. (53)] can be efficiently solved numerically.

Now we want to compute λ in the presence of the solitons and KLS walls accompanying the HQVs in the PdB phase. The soliton solutions in 1D have an analytical form and the potential is written in terms of $\theta(x)$ that satisfies the equations of motion [Supplementary Eq. (24)] as

$$U(\theta) = -2\tilde{f}_{\rm so}(\theta) + \tfrac{3}{2}(1-q_1)q_2\sin\theta - q_1 - \tfrac{1}{4}q_2^2. \tag{55}$$

The eigenvalue equation

$$\Psi_+''(x) + U(\theta)\Psi_+(x) = \lambda\Psi_+(x) \tag{56}$$

can be numerically solved for the lowest lying eigenvalue for the spin wave $\Psi_+(x)$ localized on an infinite 1D soliton. We obtain eigenvalues $\lambda(q)$ in Supplementary Figure 6 for the NMR satellite peaks with the relative NMR shifts $\Delta\omega = \lambda(q)\Omega_{\rm PdB}^2/(2\omega_{\rm L})$. The fit to the temperature dependence of $q(T/T_{\rm c})$ in Supplementary Figure 5 then leads to the values $\lambda(T/T_{\rm c})$ shown in the main text.

The experimentally measured NMR intensity relative to the bulk is given as (where the total magnetization per mode is I_M)[24–26]

$$I_M/I_0 \propto \tfrac{1}{2}n_{\rm HQV}\frac{|\int dA\, \delta S_+(x,y)|^2}{\int dA\, |\delta S_+(x,y)|^2} \tag{57}$$

where $\delta S_+(x,y) = e^{-i\theta(x)}\Psi_+(x)$ is the physical transverse spin wave excitation and $n_{\rm HQV}$ is the areal density of HQVs. These NMR oscillator intensities are shown in Supplementary Figure 7.

SUPPLEMENTARY REFERENCES

[1] N. Zhelev, T. S. Abhilash, E. N. Smith, R. G. Bennett, X. Rojas, L. Levitin, J. Saunders, and J. M. Parpia, “The A-B transition in superfluid helium-3 under confinement in a thin slab geometry,” Nature Communications **8**, 15963 (2017).

[2] L. V. Levitin, R. G. Bennett, A. Casey, B. Cowan, J. Saunders, D. Drung, T. Schurig, and J. M. Parpia, “Phase diagram of the topological superfluid ^{3}He confined in a nanoscale slab geometry,” Science **340**, 841–844 (2013).

[3] J. I. A. Li, A. M. Zimmerman, J. Pollanen, C. A. Collett, W. J. Gannon, and W. P. Halperin, “Stability of superfluid $^3\mathrm{He}-\mathrm{B}$ in compressed aerogel,” Phys. Rev. Lett. **112**, 115303 (2014).

[4] J. J. Wiman and J. A. Sauls, “Superfluid phases of ^{3}He in a periodic confined geometry,” Journal of Low Temperature Physics **175**, 17–30 (2014).

[5] V. V. Dmitriev, A. A. Senin, A. A. Soldatov, and A. N. Yudin, “Polar phase of superfluid $^3\mathrm{He}$ in anisotropic aerogel,” Phys. Rev. Lett. **115**, 165304 (2015).

[6] R. S. Askhadullin, V. V. Dmitriev, D. A. Krasnikhin, P. N. Martynov, A. A. Osipov, A. A. Senin, and A. N. Yudin, “Phase diagram of superfluid ^{3}He in “nematically ordered” aerogel,” JETP Letters **95**, 326–331 (2012).

[7] S. Yang and R. Ikeda, “Possibility of unconventional pairing states in superfluid ^{3}He in uniaxially anisotropic aerogels,” Journal of the Physical Society of Japan **83**, 084602 (2014).

[8] T. Mizushima, Y. Tsutsumi, M. Sato, and K. Machida, “Symmetry protected topological superfluid ^{3}He-B,” Journal of Physics: Condensed Matter **27**, 113203 (2015).

[9] I. A. Fomin, “Analog of Anderson theorem for the polar phase of liquid 3He in nematic aerogel,” Preprint at http://arXiv.org/abs/1803.03804 (2018).

[10] V. P. Mineyev and G. E. Volovik, “Planar and linear solitons in superfluid ^{3}He,” Phys. Rev. B **18**, 3197–3203 (1978).

[11] D. Vollhardt and P. Wölfle, *The Superfluid Phases of Helium 3* (Taylor & Francis, 1990).

[12] V. V. Dmitriev, A. A. Senin, A. A. Soldatov, E. V. Surovtsev, and A. N. Yudin, “B phase with polar distortion in superfluid ^{3}He in “ordered” aerogel,” Journal of Experimental and Theoretical Physics **119**, 1088–1096 (2014).

[13] G. E. Volovik, “Half quantum vortices in the B phase of superfluid ^{3}He,” JETP Letters **52**, 358–363 (1990).

[14] M. M. Salomaa and G. E. Volovik, “Cosmiclike domain walls in superfluid ^{3}He-B: Instantons and diabolical points in $(\mathbf{k}, \mathbf{r})$ space,” Phys. Rev. B **37**, 9298–9311 (1988).

[15] M. Silveri, T. Turunen, and E. Thuneberg, “Hard domain walls in superfluid $^3\mathrm{He} - B$,” Phys. Rev. B **90**, 184513 (2014).

[16] N. Nagamura and R. Ikeda, “Stability of half-quantum vortices in equal-spin pairing states of ^{3}He,” Phys. Rev. B **98**, 094524 (2018).

[17] D. Rainer and M. Vuorio, “Small objects in superfluid ^{3}He,” Journal of Physics C: Solid State Physics **10**, 3093–3106 (1977).

[18] G. E. Volovik, “Glass state of superfluid ^{3}He-A in aerogel,” JETP Letters **63**, 301–304 (1996).

[19] V. V. Dmitriev, D. A. Krasnikhin, N. Mulders, A. A. Senin, G. E. Volovik, and A. N. Yudin, “Orbital glass and spin glass states of ^{3}He-A in aerogel,” JETP Letters **91**, 599–606 (2010).

[20] J. I. A. Li, J. Pollanen, A. M. Zimmerman, C. A. Collett, G. W. J., and W. P. Halperin, “The superfluid glass phase of ^{3}He-A,” Nature Physics **9**, 775–779 (2013).

[21] S. Autti, V. V. Dmitriev, J. T. Mäkinen, A. A. Soldatov, G. E. Volovik, A. N. Yudin, V. V. Zavjalov, and V. B. Eltsov, “Observation of half-quantum vortices in topological superfluid ^{3}He,” Phys. Rev. Lett. **117**, 255301 (2016).

[22] V. V. Zavjalov, “Linear NMR in the polar phase of ^{3}He in aerogel,” JETP Letters **108**, 402–408 (2018), 10.1134/S0021364018180029.

[23] H. Choi, J. P. Davis, J. Pollanen, T. M. Haard, and W. P. Halperin, “Strong coupling corrections to the Ginzburg-Landau theory of superfluid ^{3}He,” Phys. Rev. B **75**, 174503 (2007).

[24] M. M. Salomaa and G. E. Volovik, “Half-quantum vortices in superfluid $^3\mathrm{He}$-A,” Phys. Rev. Lett. **55**, 1184–1187 (1985).

[25] M. M. Salomaa and G. E. Volovik, “Quantized vortices in superfluid ^{3}He,” Rev. Mod. Phys. **59**, 533–613 (1987).

[26] C.-R. Hu and K. Maki, “Satellite magnetic resonances of a bound pair of half-quantum vortices in rotating superfluid $^3He - A$,” Phys. Rev. B **36**, 6871–6880 (1987)
.
